# Supplementary material for: SARS-CoV-2 antibody immunoassays in serial samples reveal earlier seroconversion in acutely ill COVID-19 patients developing ARDS
Source: PLoS One. 2021 May 13;16(5):e0251587. doi: 10.1371/journal.pone.0251587 (PMC8118560; doi:10.1371/journal.pone.0251587)
Supplement: S1 Fig — The dotted line represents a p-value of 0.05. (PDF) [file pone.0251587.s001.pdf]

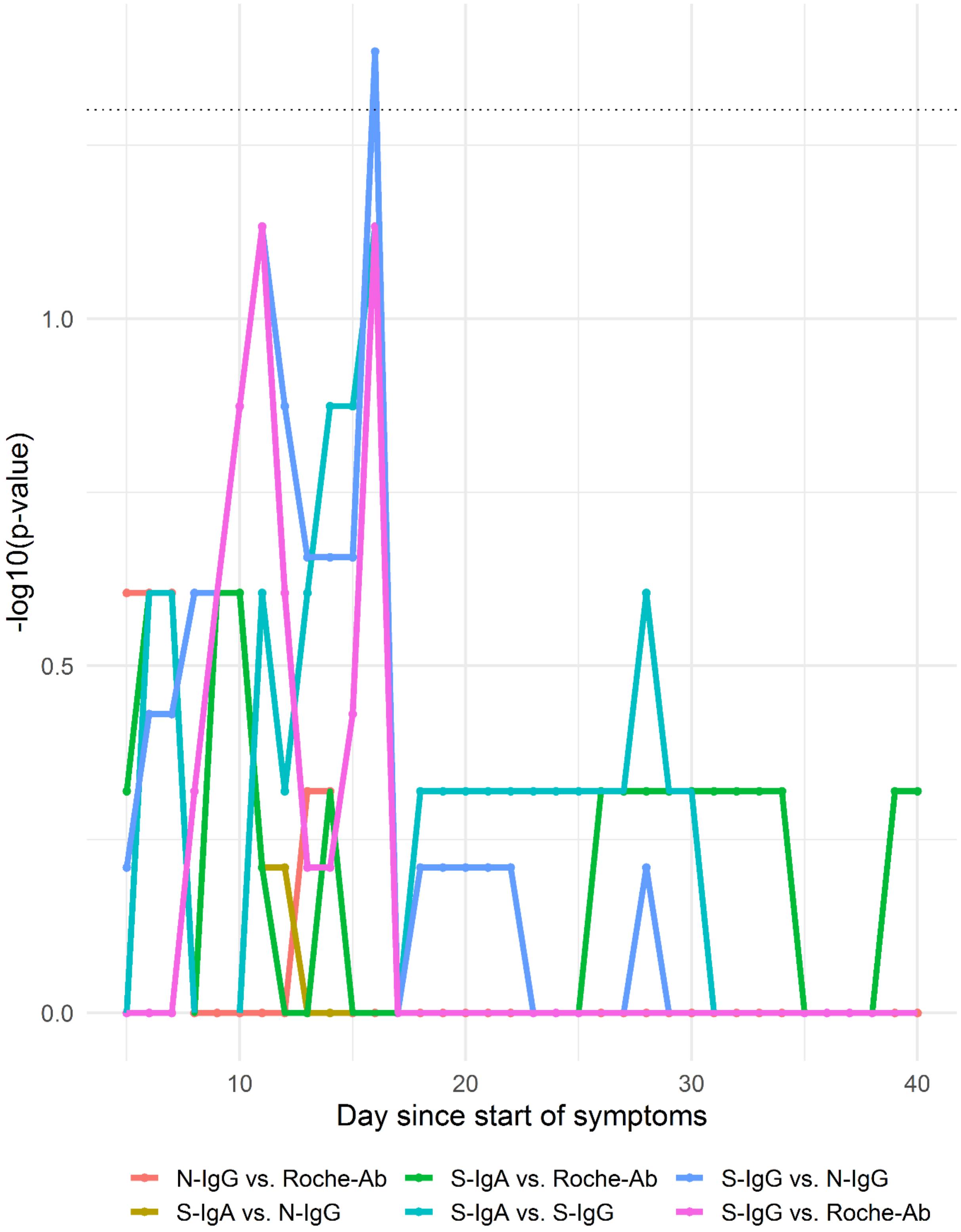

S1 Fig. p-values for differences in qualitative test results for the four immunoassays in the PCR-positive clinical cohort. The dotted line represents a p-value of 0.05.
