# Supplementary material for: SARS-CoV-2 antibody immunoassays in serial samples reveal earlier seroconversion in acutely ill COVID-19 patients developing ARDS
Source: PLoS One. 2021 May 13;16(5):e0251587. doi: 10.1371/journal.pone.0251587 (PMC8118560; doi:10.1371/journal.pone.0251587)
Supplement: S3 Fig — Spearman correlation coefficient (R) and p-values are shown. The dotted lines represent the cutoff values for a positive test result. (PDF) [file pone.0251587.s003.pdf]

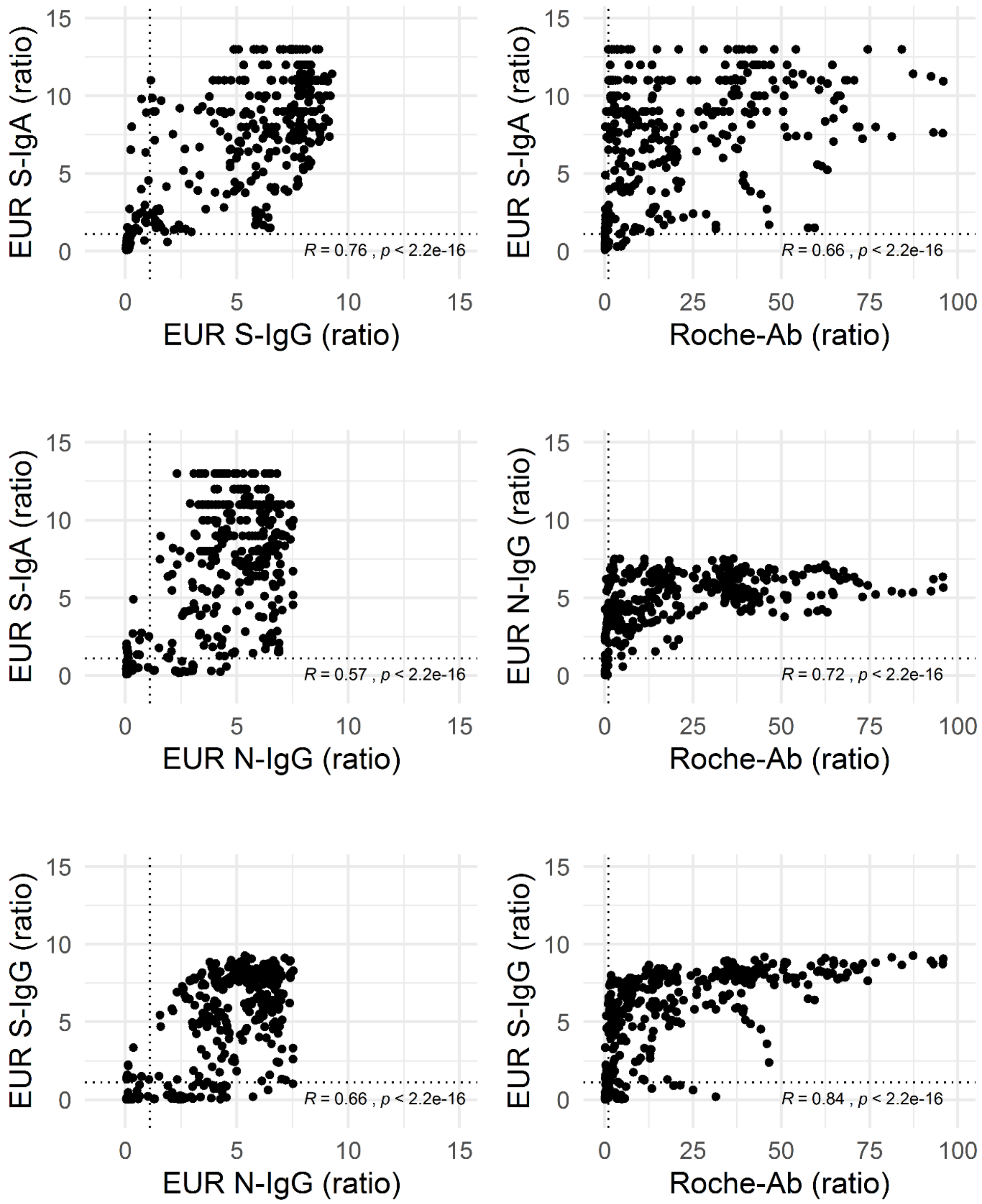

S3 Fig. Pairwise comparison of signal ratios between the different immunoassays in the PCR-positive clinical cohort. Spearman correlation coefficient (R) and p-values are shown. The dotted lines represent the cutoff values for a positive test result.
