# Supplementary material for: SARS-CoV-2 antibody immunoassays in serial samples reveal earlier seroconversion in acutely ill COVID-19 patients developing ARDS
Source: PLoS One. 2021 May 13;16(5):e0251587. doi: 10.1371/journal.pone.0251587 (PMC8118560; doi:10.1371/journal.pone.0251587)
Supplement: S6 Fig — (PDF) [file pone.0251587.s006.pdf]

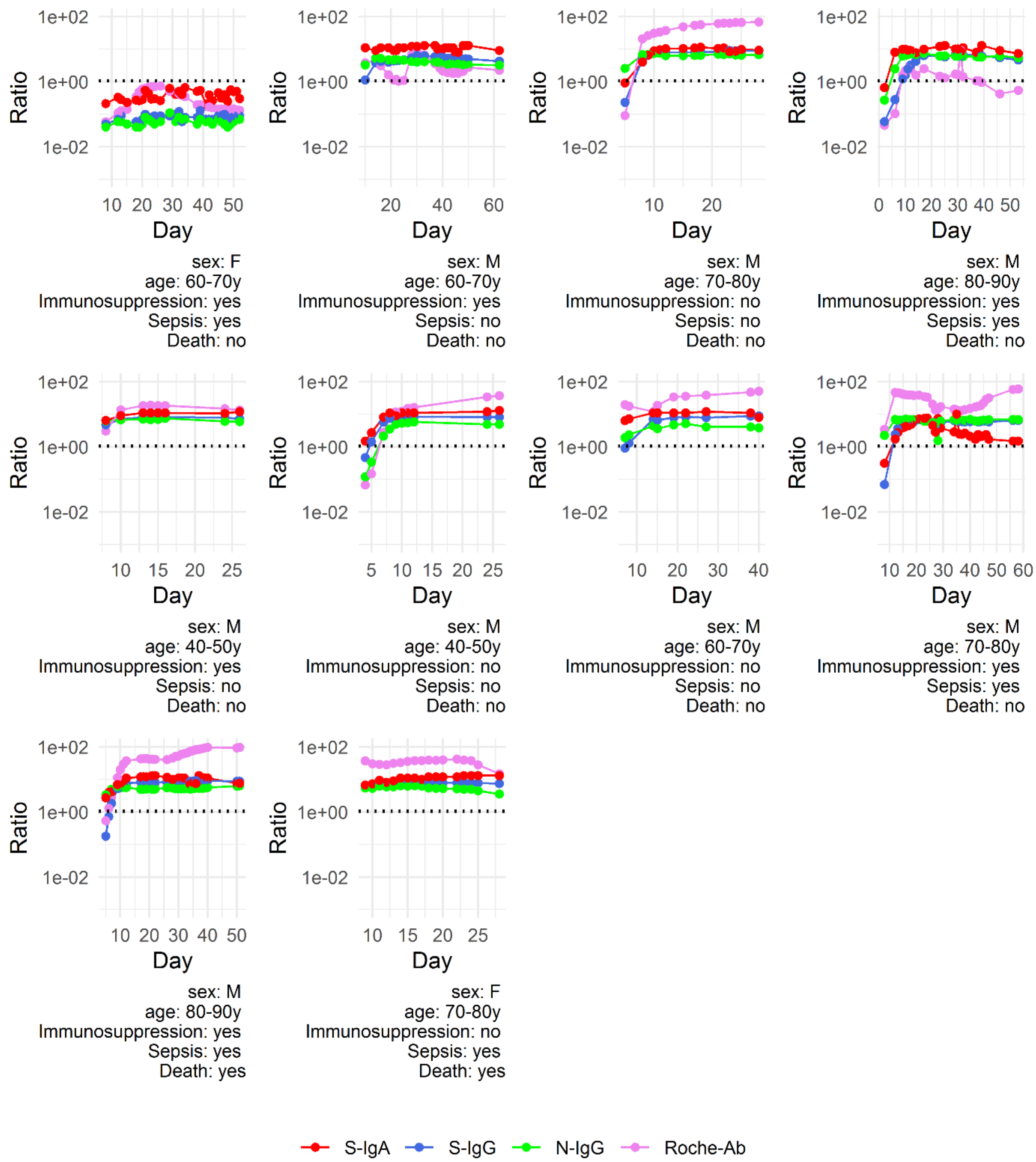

S6 Fig. Individual results in the PCR-positive clinical cohort for the four different immunoassays in the ARDS group (first set).
