# Supplementary material for: SARS-CoV-2 antibody immunoassays in serial samples reveal earlier seroconversion in acutely ill COVID-19 patients developing ARDS
Source: PLoS One. 2021 May 13;16(5):e0251587. doi: 10.1371/journal.pone.0251587 (PMC8118560; doi:10.1371/journal.pone.0251587)
Supplement: S11 Fig — (A) Pre-COVID-19 cohort. (B) PCR-negative clinical cohort. (This plot was generated using the UpSetR R package). (PDF) [file pone.0251587.s011.pdf]

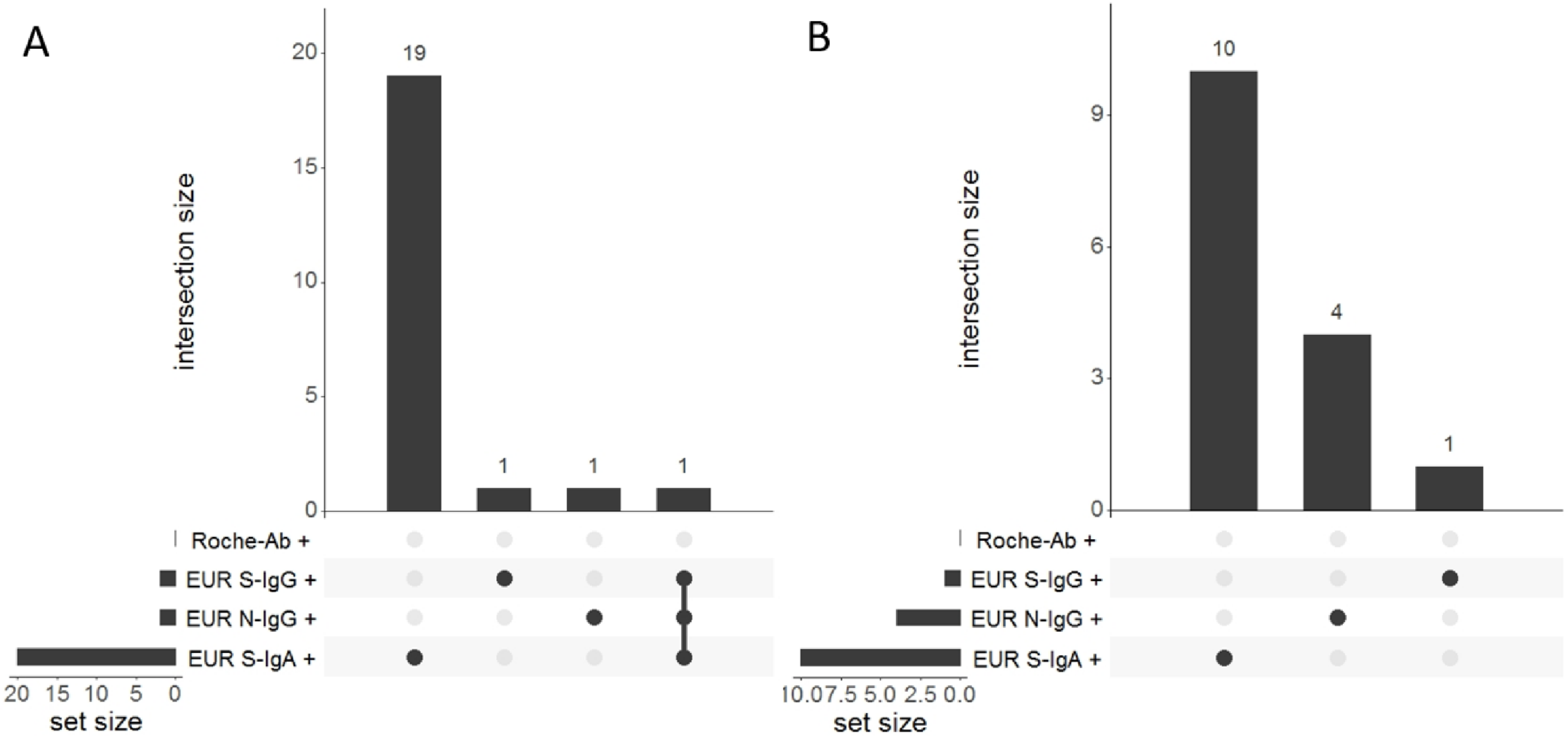

S11 Fig. Overlap of positive results between immunoassays in the two negative cohorts. (A) Pre-COVID-19 cohort. (B) PCR-negative clinical cohort.

(This plot was generated using the UpSetR R package) (1)

References

1. Jake R Conway, Alexander Lex, Nils Gehlenborg UpSetR: An R Package for the Visualization of Intersecting Sets and their Properties
doi: https://doi.org/10.1093/bioinformatics/btx364
