# Supplementary material for: SARS-CoV-2 antibody immunoassays in serial samples reveal earlier seroconversion in acutely ill COVID-19 patients developing ARDS
Source: PLoS One. 2021 May 13;16(5):e0251587. doi: 10.1371/journal.pone.0251587 (PMC8118560; doi:10.1371/journal.pone.0251587)
Supplement: S12 Fig — The dotted lines represent the cutoff values for a positive test result. (A) EUR S-IgA. (B) EUR S-IgG. (C) EUR N-IgG. (D) Roche-Ab. (PDF) [file pone.0251587.s012.pdf]

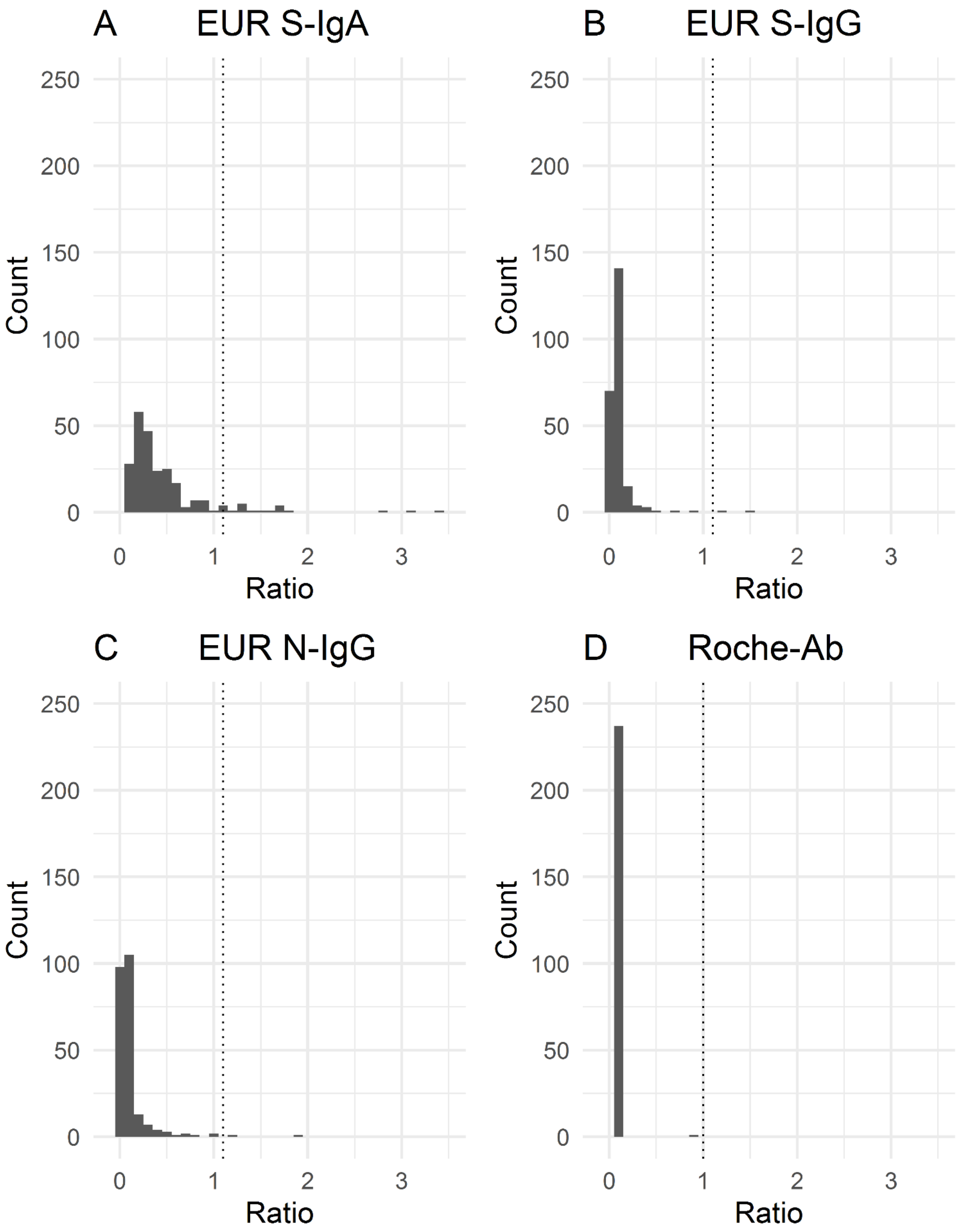

S12 Fig. Distributions of signal ratios for the four different immunoassays in the pre-COVID-19 cohort. The dotted lines represent the cutoff values for a positive test result. (A) EUR S-IgA. (B) EUR S-IgG. (C) EUR N-IgG. (D) Roche-Ab.
