# Supplementary material for: SARS-CoV-2 antibody immunoassays in serial samples reveal earlier seroconversion in acutely ill COVID-19 patients developing ARDS
Source: PLoS One. 2021 May 13;16(5):e0251587. doi: 10.1371/journal.pone.0251587 (PMC8118560; doi:10.1371/journal.pone.0251587)
Supplement: S1 Table — (PDF) [file pone.0251587.s014.pdf]

S1 Table. Sensitivities (with 95% confidence interval) of the different immunoassays grouped into time bins.

time period	n	EUR S-IgA	EUR S-IgG	EUR N-IgG	Roche-Ab
<5	10	10.0 (0.3% to 44.5%)	10.0 (0.3% to 44.5%)	20.0 (2.5% to 55.6%)	0.0 (0.0% to 30.8%)
5-9	24	50.0 (29.1% to 70.9%)	50.0 (29.1% to 70.9%)	62.5 (40.6% to 81.2%)	62.5 (40.6% to 81.2%)
10-14	28	78.6 (59.0% to 91.7%)	64.3 (44.1% to 81.4%)	78.6 (59.0% to 91.7%)	71.4 (51.3% to 86.8%)
15-19	26	88.5 (69.8% to 97.6%)	80.8 (60.6% to 93.4%)	88.5 (69.8% to 97.6%)	84.6 (65.1% to 95.6%)
20-29	28	96.4 (81.7% to 99.9%)	89.3 (71.8% to 97.7%)	96.4 (81.7% to 99.9%)	92.9 (76.5% to 99.1%)
30-39	13	92.3 (64.0% to 99.8%)	92.3 (64.0% to 99.8%)	92.3 (64.0% to 99.8%)	92.3 (64.0% to 99.8%)
40-49	9	88.9 (51.8% to 99.7%)	88.9 (51.8% to 99.7%)	88.9 (51.8% to 99.7%)	77.8 (40.0% to 97.2%)
50-64	8	87.5 (47.3% to 99.7%)	87.5 (47.3% to 99.7%)	75.0 (34.9% to 96.8%)	62.5 (24.5% to 91.5%)
