# Supplementary material for: SARS-CoV-2 antibody immunoassays in serial samples reveal earlier seroconversion in acutely ill COVID-19 patients developing ARDS
Source: PLoS One. 2021 May 13;16(5):e0251587. doi: 10.1371/journal.pone.0251587 (PMC8118560; doi:10.1371/journal.pone.0251587)
Supplement: S2 Table — (PDF) [file pone.0251587.s015.pdf]

S2 Table. Median age for true negative and false positive subjects in the negative cohorts.

assay	test result	median age pre-COVID19 cohort	median age PCR-negative clinical cohort	median age combined	p-value
**EUR S-IgA**	neg.	27	70	49	0.0294
	pos.	28	66	30	
**EUR S-IgG**	neg.	27	69	48	0.7660
	pos.	41	73	53	
**EUR N-IgG**	neg.	27	69	47	0.0253
	pos.	31	93	89	
**Roche-Ab**	neg.	27	69	48	/
	pos.	/	/	/	
